# Reversible complete atrioventricular block caused by aortic bicuspid valve calcification with severe aortic stenosis: a case report

**DOI:** 10.1093/ehjcr/ytae173

**Published:** 2024-04-09

**Authors:** Shuichiro Yamauchi, Hidekazu Kondo, Tomoko Fukuda, Shinji Miyamoto, Naohiko Takahashi

**Affiliations:** Department of Cardiology and Clinical Examination, Faculty of Medicine, Oita University, 1-1 Idaigaoka, Hasama, Yufu City, 879-5593 Oita, Japan; Department of Cardiology and Clinical Examination, Faculty of Medicine, Oita University, 1-1 Idaigaoka, Hasama, Yufu City, 879-5593 Oita, Japan; Department of Cardiology and Clinical Examination, Faculty of Medicine, Oita University, 1-1 Idaigaoka, Hasama, Yufu City, 879-5593 Oita, Japan; Department of Cardiovascular Surgery, Faculty of Medicine, Oita University, Yufu City, Oita, Japan; Department of Cardiology and Clinical Examination, Faculty of Medicine, Oita University, 1-1 Idaigaoka, Hasama, Yufu City, 879-5593 Oita, Japan

**Keywords:** Case report, Reversible complete atrioventricular block, Aortic stenosis, Bicuspid aortic valve, Electrocardiogram-gated computed tomography, ^18^F-fluorodeoxyglucose-positron emission tomography

## Abstract

**Background:**

The aetiology of secondary complete atrioventricular blocks includes ischaemia, cardiac sarcoidosis, electrolyte imbalance, drug use, rheumatic fever, and infections such as Lyme disease and endocarditis. Diagnosis is important since some of these causes are reversible. Although several studies have reported on aortic valve calcification causing complete atrioventricular blocks, no study has described improvement of complete atrioventricular blocks by removal of the calcification.

**Case summary:**

A 42-year-old man with syncope had a Mobitz type II atrioventricular block, an alternating bundle branch block, and severe aortic stenosis. We identified a 10 s paroxysmal complete atrioventricular block with pre-syncope and performed pacemaker implantation. Electrocardiography-gated computed tomography confirmed that the calcification had reached the muscular septum. ^18^F-fluorodeoxyglucose-positron emission tomography (FDG-PET) revealed significant FDG uptake with high CT value of calcification in basal interventricular septum. The calcification in the septum was removed carefully, and aortic valve replacement was performed. The atrioventricular conduction capacity improved post-surgery. During the 1-year follow-up, the patient reported dramatic improvement in exercise capacity. We also noted an improvement of <0.1% in the right ventricular pacing burden.

**Discussion:**

Complete atrioventricular blocks occur in patients with aortic stenosis accompanied by severe calcification of the aortic valve, which are visualized comprehensively by echocardiography. Electrocardiography-gated computed tomography and FDG-PET enabled detailed evaluation of the extent of calcification and pre- and post-operative tissue inflammation. Hence, we suspected that the calcification in the septum was causing complete atrioventricular block. Moreover, clinicians should recognize that aortic valve calcification with aortic stenosis can cause complete atrioventricular blocks.

**Learning points**

- To remember that complete atrioventricular block (CAVBs) may be caused by aortic valve calcification associated with aortic stenosis.
- To confirm the utility of electrocardiography-gated CT and ^18^F-fluorodeoxyglucose-positron emission tomography in elucidating the pathology of CAVBs associated with aortic valve calcification.
- To consider whether pacemaker implantation or aortic valve replacement should be prioritized based on the patient’s current situation and background.

## Introduction

The aetiology of secondary complete atrioventricular block (CAVB) includes ischaemia, cardiac sarcoidosis, electrolyte imbalance, drug use, rheumatic fever, and infections such as Lyme disease and endocarditis.^1,2^ Diagnosis is important, especially in younger patients, since some of these causes are reversible. Although several studies have reported on CAVB caused by aortic stenosis (AS) with calcification,^3,4^ no study has described the improvement in CAVB after removal of the calcification. In this study, we report a case where AS calcification was confirmed to extend into the septum by imaging modalities, the removal of which improved atrioventricular conduction.

## Summary figure

Schematic of the manner in which aortic valve calcification impairs atrioventricular conduction

The bundle of His and left bundle branch run close to the base of the commissure between the NCC and RCC. Calcification between the NCC and RCC can damage the bundle of His to left bundle branch due to compression and inflammation.

LCC, left coronary cusp; NCC, non-coronary cusp; RCC, right coronary cusp

**Figure:**
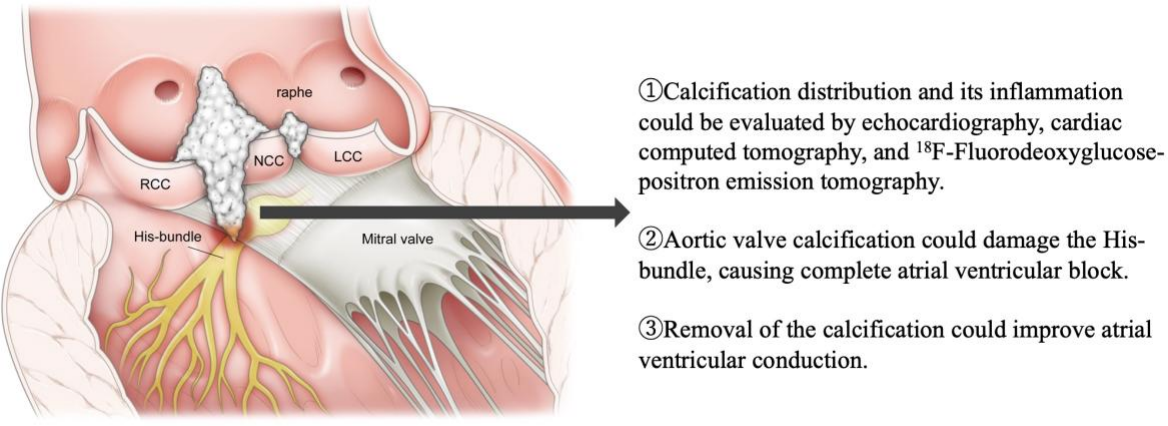


## Case summary

**Figure 1:**
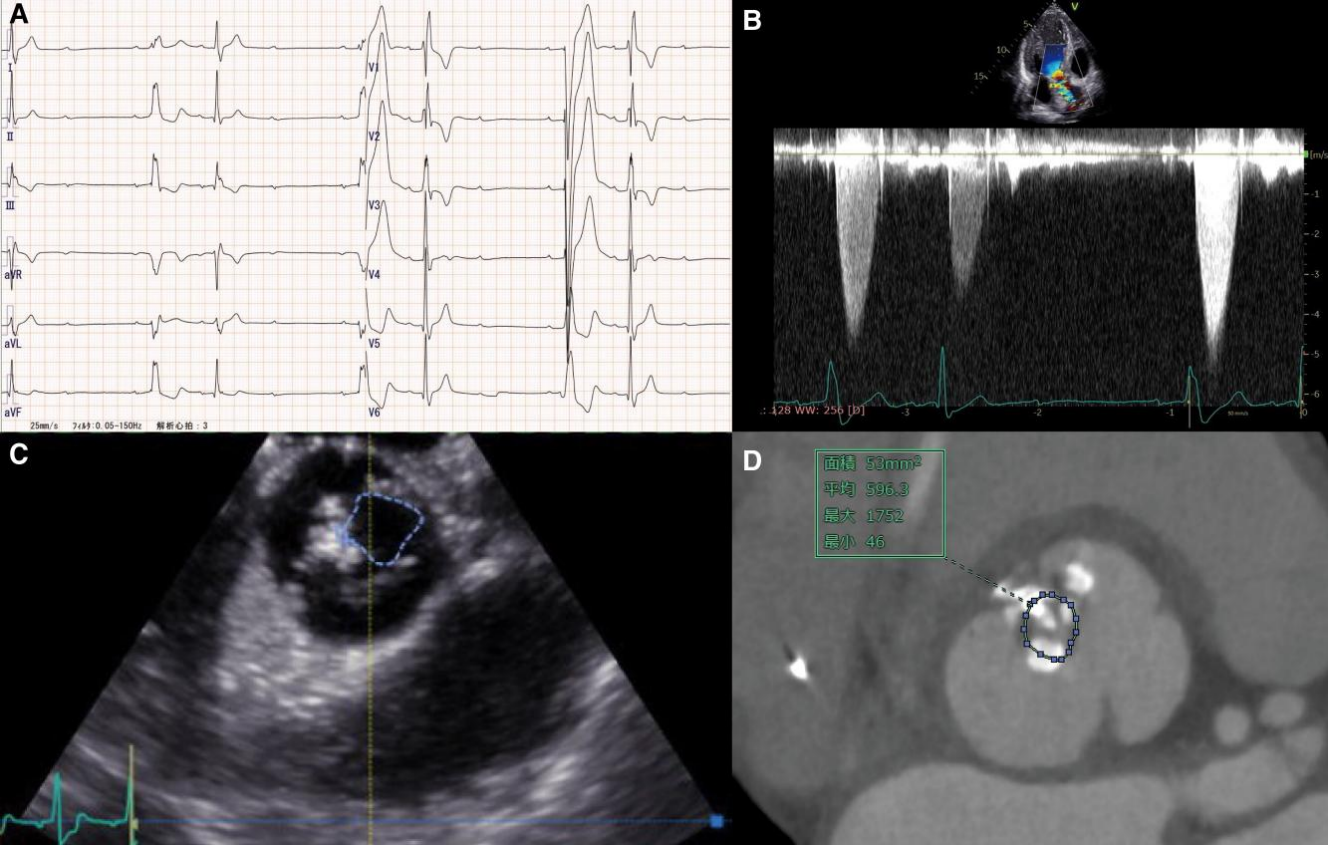
(*A*) Electrocardiography showing a Mobitz type II atrioventricular block and alternating bundle branch block. (*B*) Transthoracic echocardiography showing a maximum aortic valve flow velocity of 5.0 m/s at long RR intervals. (*C*) Three-dimensional transoesophageal echocardiography showing a bicuspid aortic valve with an area of 0.86 cm^2^. (*D*) Electrocardiography-gated CT at mid-systole showing a bicuspid aortic valve with area of 0.53 cm^2^. CT, computed tomography.

**Figure 2:**
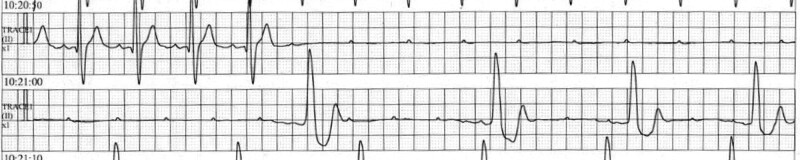
Electrocardiography showing a paroxysmal atrioventricular block with pre-syncope.

**Figure 3:**
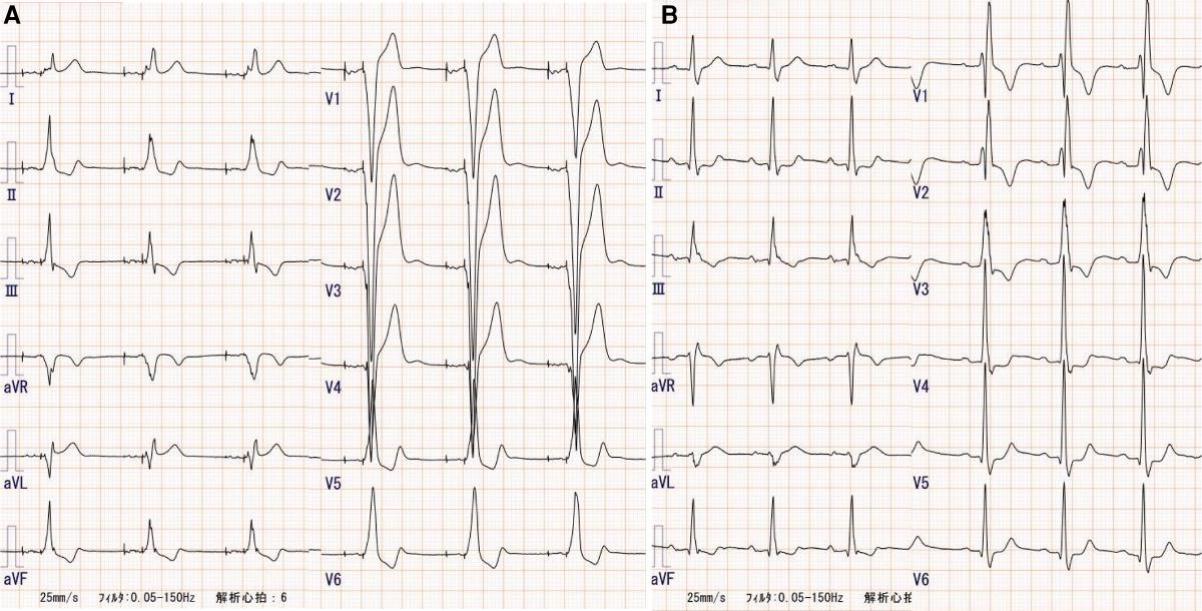
Electrocardiography acquired before (*A*) and after (*B*) surgery.

**Figure 4:**
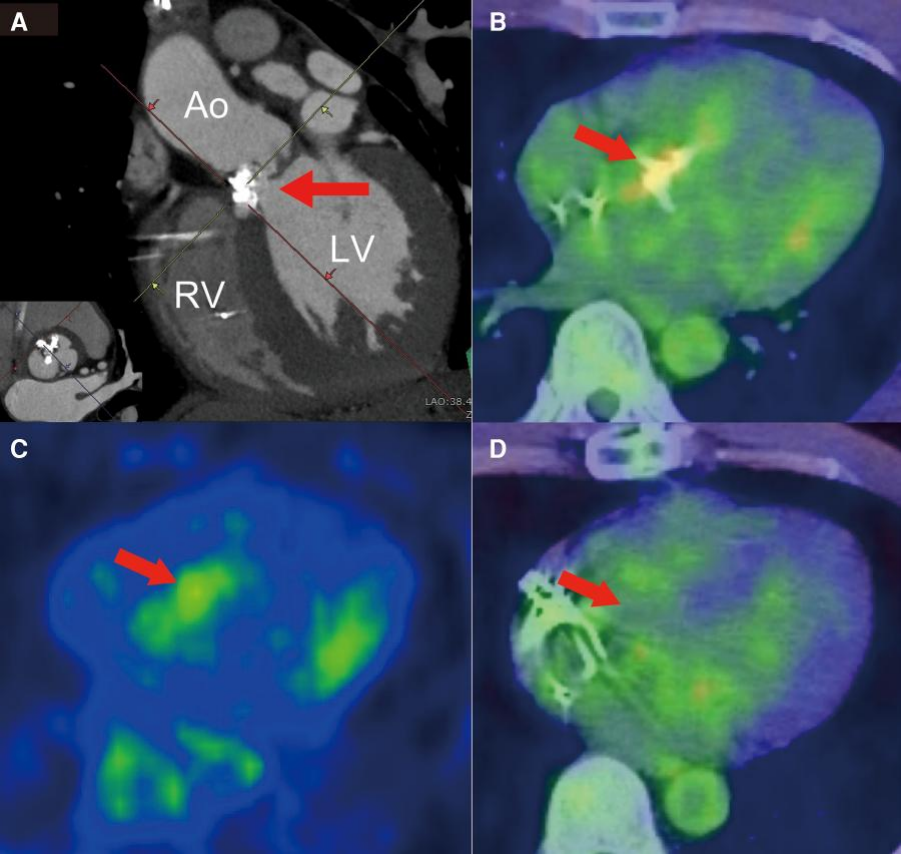
(*A*) Electrocardiography-gated computed tomography (CT) image revealing calcification reaching the muscular septum (arrow). (*B*) FDG-PET image showing accumulation of FDG in the aortic valve and calcified lesions connected to the interventricular septum (arrow). (*C*) FDG-PET only images of *panel B*. The maximum standardized uptake value at the arrow is 3.53. (*D*) FDG-PET imaging after surgery showing an obvious reduction of FDG uptake in the aortic valve and basal interventricular septum (arrow). Ao, aorta; LCC, left coronary cusp; LV, left ventricle; NCC, non-coronary cusp; RCC, right coronary cusp; RV, right ventricle; FDG-PET, fluorodeoxyglucose-positron emission tomography.

A 42-year-old Asian man who experienced syncope for a few seconds was admitted to our hospital. He had a habit of strength training, during which he had recently begun to faint frequently. No other symptoms were noted. He had no previous syncope and relevant medical history. The patient’s mother had cardiac sarcoidosis that was treated with corticosteroids and cardiac resynchronization therapy with a defibrillator. His father died suddenly at the age of 30 years due to unknown causes. Physical examination showed a systolic ejection murmur and an irregular pulse. No other relevant findings were found in the physical examination. Laboratory tests showed no abnormal findings, and the value of NT-proBNP was 26.2 pg/mL. Electrocardiography revealed sinus rhythm with a Mobitz type II atrioventricular block pattern and an alternating bundle branch block (*Figure 1A*). Transthoracic and transoesophageal echocardiography revealed severe AS caused by a bicuspid aortic valve (BAV) (*Figure 1B* and *C*), albeit left ventricular ejection fraction 63% with normal wall motion and normal diastolic function, without morphological abnormalities or other valvular disease. On the second day of admission, the patient experienced pre-syncope, and monitoring documented a paroxysmal atrioventricular block with 10 s of ventricular arrest, followed by a transient complete atrioventricular block (*Figure 2*). Permanent pacemaker implantation was performed on Day 4 of admission. Subsequently, electrocardiography showed atrial and ventricular pacing rhythms (*Figure 3A*), and the right ventricular pacing burden exceeded 99.9%. Electrocardiography-gated computed tomography (CT) confirmed that the calcification extended to the muscular septum (*Figure 4A*). The aortic valve area measured from CT was 0.53 cm^2^, indicating severe AS (*Figure 1D*). ^18^F-fluorodeoxyglucose-positron emission tomography (^18^F-FDG-PET) revealed significant FDG uptake with high CT value due to calcification in the aortic valve and basal interventricular septum, which showed maximum standardized uptake value was 3.53 (*Figure 4B* and *C*). The patient had a family history of cardiac sarcoidosis and was therefore examined in detail for sarcoidosis. No lesions in other organs, such as the eyes or lungs, were suspicious of sarcoidosis. Serum angiotensin converting enzyme, lysozyme, and soluble interleukin 2 receptor were all within normal limits. A cardiac MRI was also performed before pacemaker implantation, but there were no myocardial oedema or late gadolinium enhancement. These findings (only two positive findings: positive FDG-PET and advanced atrioventricular block) did not meet the diagnostic criteria for JCS 2016 Guideline on Diagnosis and Treatment of Cardiac Sarcoidosis.^5^ Exercise echocardiography showed an increase in aortic valve blood flow velocity from 4.4 m/s to 5.4 m/s and an increase in mean systolic pressure gradient from 38.4 mmHg to 67.0 mmHg (see Supplementary material-online, *Table S1*). Although findings corresponding to severe AS were observed, the patient had no hypotension or symptoms, indicating severe asymptomatic AS. Despite the patient presenting with asymptomatic AS, we considered aortic valve surgery for several reasons. First, there was a possibility of CAVB, possibly due to calcification and inflammation of that calcification, and removal of the calcification could have improved the CAVB; second, the patient was physically very active; and third, exercise stress echocardiography showed an increase in mean aortic valve pressure gradient of more than 20 mmHg, an indicator of poor prognosis.^6^ Finally, we explained the risks, complications, and benefits of the surgery, and the patient was willing to undergo aortic valve surgery. Two months later, we performed surgical replacement of the aortic valve. The type 1 BAV showed fusion of the left coronary and non-coronary cusps, and calcification between the right and non-coronary cusps reaching the muscular septum. The calcification in the septum was carefully removed and aortic valve replacement (AVR) was performed. We implanted ATS bi-leaflet mechanical valve with a size of 20 mm because the patient was young. Notably, the atrioventricular conduction capacity improved remarkably (i.e. normal sinus rhythm with complete right bundle branch block, *Figure 3B*) by the 14th post-operative day. Three months after surgery, ^18^F-FDG-PET revealed a remarkable reduction in the basal intraventricular uptake (*Figure 4D*). The patient was discharged on the 16th post-operative day without complications. During the 1-year follow-up period, the patient reported a dramatic improvement in exercise capacity. We also noted an improvement of <0.1% in the right ventricular pacing burden.

## Discussion

To the best of our knowledge, this is the first study to report that ^18^F-FDG-PET and CT revealed that aortic valve calcification with severe AS reached the cardiac conduction system near the bundle of His, causing inflammation, and leading to CAVB (*Summary figure*). Furthermore, in this case, AVR with removal of the calcification improved atrioventricular conduction, and post-operative ^18^F-FDG-PET showed amelioration of inflammation in the cardiac conduction system.

Studies have been reported that CAVB occurs in patients with AS accompanied by severe calcification of the aortic valve.^3,4^ Reza *et al*. reported a case in which calcification invaded the interventricular septum, which was visualized in detail by echocardiography.^4^ Electrocardiography-gated cardiac CT has high spatial resolution in the assessment of calcification compared to all other imaging modalities.^7,8^ 3D multiplanar reconstruction techniques allow accurate evaluation of cardiac structures, including the severity, location, and extent of calcification. Aortic stenosis generally results from an active disease process similar to atherosclerosis with lipoprotein deposition, chronic inflammation, and active leaflet calcification,^9^ and FDG-PET visualizes that inflammation. Marincheva *et al*.^10^ reported that AS with higher aortic valve FDG-PET signals was more likely to have progressive stenosis, which might help to determine shorter follow-up intervals and the timing of surgery. Furthermore, FDG-PET is required to assess the cardiac sarcoidosis as a cause of reversible CAVB.^5^ In this case, electrocardiography-gated CT or FDG-PET imaging enabled more detailed evaluation of the extent of calcification and the pre- and post-operative tissue inflammation (*Figure 4*). These findings led us to suspect that the calcification on the septum was causing CAVB. In addition, an increase in aortic valve mean pressure gradient of more than 20 mmHg with exercise and a severe aortic valve calcification were indicators of poor prognosis, which provided a strong indication to perform AVR.^6^ Ruling out cardiac sarcoidosis was one of the most important aspects of this case. There were only two findings suggestive of cardiac sarcoidosis in this patient. Furthermore, it is unlikely that the patient had cardiac sarcoidosis, given the loss of FDG uptake on post-operative FDG-PET images. However, the patient should be followed up for future development of familial sarcoidosis.

Most importantly, it was necessary to verify whether the order of treatment (pacemaker implantation preceding AVR) was appropriate. In retrospect, we believe that permanent pacemaker implantation could have been avoided if calcification removal and AVR had been performed first. Although pacemaker implantation for CAVB due to transient causes is not recommended in the 2021 ESC Guidelines on cardiac pacing and cardiac resynchronization therapy,^2^ there was no way to prove that atrioventricular conduction was reversible in the pre-operative setting. Moreover, as mentioned earlier, the syncope symptoms that troubled the patient most were caused by severe bradycardia. Aortic stenosis was also severe but asymptomatic and did not require urgent AVR. In addition, some patients presented with severe atrioventricular block in the subacute to chronic phase after aortic valve replacement, and permanent pacemaker implantation was performed first. Based on the findings of this case, AVR and septal calcification removal first may be an option if prompt surgery is possible. We hope that more evidence will be established through the accumulation of similar cases in the future.

## Conclusion

Herein, we report a case of reversible CAVB associated with aortic valve calcification. Clinicians should recognize that aortic valve calcification with AS can cause CAVB. Further studies are needed to determine whether pacemaker implantation or aortic valve replacement should be prioritized.

## Supplementary Material

ytae173_Supplementary_Data

## Lead author biography

Shuichiro Yamauchi graduated from Oita University and completed his MD in 2013. Thereafter, he completed his cardiological fellowship at Beppu Medical Centre and Oita Medical Centre. He is currently working as a cardiologist at the Department of Cardiology of Oita University of Oita in Japan. His academic interests include structural heart disease and interventional cardiology.

## Supplementary material

**Consent:** The authors confirmed that written consent for submission and publication of this case report including images and associated text has been obtained from the patient in line with COPE guidance.

**Data availability:** The data underlying this article are available in the article and in its online Supplementary material.
