# Supplementary material for: Reversible complete atrioventricular block caused by aortic bicuspid valve calcification with severe aortic stenosis: a case report
Source: Eur Heart J Case Rep. 2024 Apr 9;8(4):ytae173. doi: 10.1093/ehjcr/ytae173 (PMC11020220; doi:10.1093/ehjcr/ytae173)
Supplement: ytae173_Supplementary_Data [file ytae173_supplementary_data.docx]

**Supplemantary material**

**Table 1: Results of the exercise stress echocardiography using an ergometer**

|  | **At rest** | **At peak exercise stress** |
| --- | --- | --- |
| **Load (W)** |  | 100 |
| **Blood pressure (mmHg)** | 131/72 | 143/61 |
| **Heart rate (bpm)** | 69 | 124 |
| **Aortic valve blood flow velocity (m/s)** | 4.4 | 5.4 |
| **Peak pressure gradient (mmHg)** | 76 | 116 |
| **Mean pressure gradient (mmHg)** | 39 | 72 |
| **Aortic valve area (cm^2^)** | 1.3 | 1.1 |
| **Aortic valve area index (cm^2^/m^2^)** | 0.7 | 0.6 |
